# Antibodies Specific for Carbamylated Proteins Precede the Onset of Clinical Symptoms in Mice with Collagen Induced Arthritis

**DOI:** 10.1371/journal.pone.0102163

**Published:** 2014-07-15

**Authors:** Jeroen N. Stoop, Bi-Sheng Liu, Jing Shi, Diahann T. S. L. Jansen, Martin Hegen, Tom W. J. Huizinga, Leendert A. Trouw, René E. M. Toes

**Affiliations:** 1 Department of Rheumatology, Leiden University Medical Center, Leiden, The Netherlands; 2 Immunoscience Research Unit, Pfizer Worldwide Research and Development, Cambridge, Massachusetts, United States of America; University Hospital Jena, Germany

## Abstract

**Objective:**

The immune response to post-translationally modified antigens is a key characteristic of rheumatoid arthritis. Carbamylation is such a posttranslational modification. Recently, we demonstrated that autoantibodies recognizing carbamylated proteins are present in sera of rheumatoid arthritis. The molecular mechanisms underlying the break of tolerance and hence the induction of anti-CarP antibody responses are unknown as well as their appearance in mouse models for systemic arthritis. Therefore we analyzed their appearance in the mouse collagen-induced arthritis model.

**Methods:**

collagen induced arthritis was induced by immunization with type II collagen in complete Freund's adjuvant. Arthritis severity was monitored by clinical scoring and anti-CarP antibody levels were determined by ELISA.

**Results:**

Anti-CarP antibodies were detectable in mice with collagen induced arthritis. We did not detect ACPA in mice with collagen induced arthritis. The specificity of the antibodies for carbamylated proteins was confirmed by inhibition assays and immunoblotting. Injection with complete Freund's adjuvant without type II collagen could also induce anti-CarP antibodies, however, in mice with arthritis, the anti-CarP antibody response was stronger and developed more rapidly. The onset of collagen induced arthritis was preceded by an increase of anti-CarP IgG2a levels in the serum.

**Conclusion:**

In mice with collagen induced arthritis we did not observe an immune response against citrullinated antigens, but we did observe an immune response against carbamylated antigens. This anti-CarP response already appeared before disease onset, indicating that collagen induced arthritis can be used as an in vivo model to study anti-CarP antibodies. Our data also indicate that the tolerance to carbamylated proteins, in contrast to the response to citrullinated proteins, is easily broken and that arthritis boosts the immune response against these proteins. The anti-CarP response in mice with CIA can be used as a model for immune responses to post-translationally modified proteins.

## Introduction

Rheumatoid arthritis (RA) is a disease characterized by a chronic inflammation of synovial joints, causing cartilage resorption and bone destruction [1]. RA affects approximately 0.5–1% of the adult population [2]. The immune response to post-translational modification of proteins is believed to play a role in the pathogenesis of RA. It is unclear how the breaking of tolerance to such modified proteins occurs. Citrullination is such a posttranslational modification. During this reaction an arginine is converted into citrulline by peptidyl arginine deiminase enzymes. This enzymatic reaction results in the generation of citrullinated antigens that are recognized by anti-citrullinated protein antibodies (ACPA; as reviewed in [3]). ACPA are diagnostic markers for rheumatoid arthritis [4] and are thought to play a role in disease pathogenesis. ACPA can activate cells and complement in vitro [5], [6]. There is discussion in the literature on whether ACPA can be detected in mice with CIA. Although some groups did detect ACPA in mice with CIA [7], [8], other groups could not [9]. Furthermore, conflicting results have been published on whether ACPA can modify disease in mice [7], [10].

**Figure 1:**
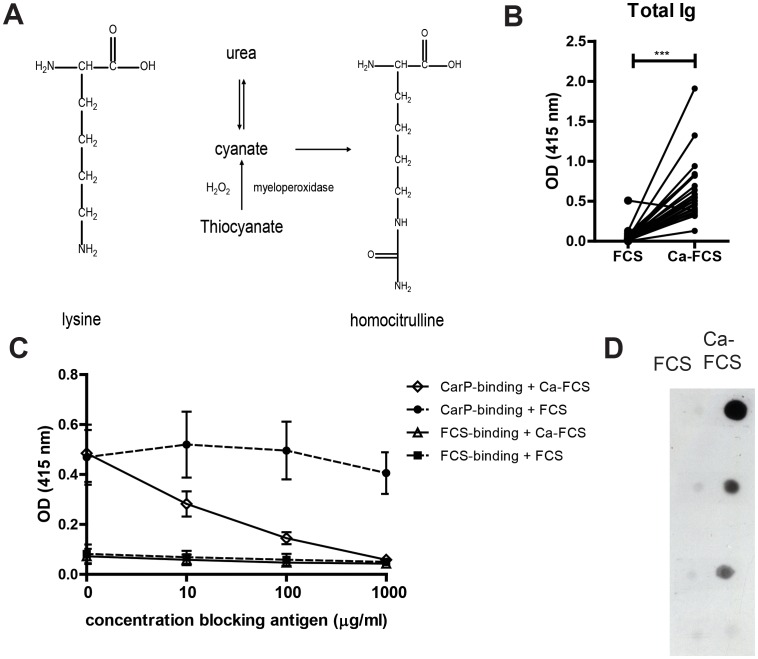
Anti-CarP antibodies can be detected in mice. (**A**) Schematic picture of the carbamylation process. (**B**) DBA/1J mice were immunized with CII in CFA. The antibody binding to FCS and Carbamylated-FCS (Ca-FCS) was determined by ELISA. The OD value for Ca-FCS binding and FCS binding of each sample are connected with a line. Statistical analysis was performed using a Wilcoxon paired test (n = 29). (**C**) Sera from anti-CarP positive mice were pre-incubated with different concentrations of Ca-FCS and FCS. The Ig binding to FCS and Ca-FCS was determined by ELISA (n = 4). (**D**) C57Bl/6 mice were immunized with CII in CFA. Equal amounts of Ca-FCS and FCS were blotted on a membrane. The presence of antibodies reactive to the Ca-FCS or FCS on the blots was analyzed by incubating the blots with sera from the immunized mice. A representative example of 2 independent experiments is depicted.

Carbamylation is a different type of post-translational modification of proteins, in which isocyanic acid reacts with the amine group of an amino acid. Carbamylation will mostly result in the conversion of lysine into homocitrulline. However, under specific conditions, other amino acids, such as arginine and cysteine and the n-terminus of a protein can also react with cyanate (as reviewed in [11]). Unlike citrullination, which is enzymatically mediated, carbamylation is a chemical reaction involving cyanate which is present in the body in equilibrium with urea. During inflammation, myeloperoxidase, which converts thiocyanate to cyanate, is released from neutrophils. This myeloperoxidase release can lead to a local increase in cyanate levels enabling further carbamylation to occur [12], [13]. A schematic picture of the carbamylation of a lysine is depicted in Figure 1A.

There is evidence for a role for carbamylation in arthritis. Recently, we identified antibodies against carbamylated proteins (anti-CarP) in the serum of RA patients and showed that the presence of these antibodies is predictive of worse disease progression in ACPA negative patients [14]. We have also shown that Anti-CarP antibodies are present in arthralgia patients and that their presence predicts the development of RA, independent of the patient's ACPA status, indicating that anti-CarP might also be useful as a biomarker for seronegative RA [15]. In mice it has been reported that intra-articular injection of a citrullinated fillagrin peptide can lead to local arthritis when the animals have previously been vaccinated with a carbamylated peptide [16].

Mouse models are frequently used tools to study the break of tolerance towards self-antigens as well as the disease pathogenesis such as occurring in arthritis. The aim of this study was to gain insight into the requirements for the emergence of anti-CarP antibody responses and to determine whether anti-CarP antibodies are also present in mice with collagen induced arthritis (CIA), the most frequently used animal model of arthritis. Here, we show for the first time that anti-CarP antibodies can be detected in mice with systemic arthritis and that the appearance of anti-CarP antibodies in the serum precedes the onset of arthritis.

## Materials and Methods

### Animals and arthritis induction

All animal experiments were performed conform national guidelines and the study protocol was approved by the Ethical Committee for Animal Experimentation (Dier Experimentele Commissie; DEC) of Leiden University. DBA/1J mice were obtained from our own breeding colony (originally obtained from Charles River). C57Bl/6 mice were purchased from Charles River. CIA was induced in 8–10 week old mice by injection at the tail base with 100 µg of CII emulsified in complete Freunds adjuvant (CFA). On day 21 the mice received a subcutaneous boost with 100 µg of CII in incomplete Freunds adjuvant (IFA; Sigma-Aldrich). For DBA/1J mice bovine CII (Chondrex) and CFA containing 0.5 mg/ml of *M.tuberculosis* (Difco) were used and for C57Bl/6 mice chicken CII (Chondrex) and CFA containing 2.5 mg/ml of *M.tuberculosis* (Chondrex) were used. For the CFA only immunizations PBS was used instead of CII. Arthritis severity was monitored as described earlier using a clinical score with a maximum of 15 per paw [17]. For the kinetics experiments, mice were bled before immunization, on day 7, 14, 21, 28, 35, 49 after immunization and at the end of follow up. Blood was centrifuged and serum was harvested and stored at −80°C until use.

### Carbamylation and citrullination of FCS

For generating carbamylated Fetal Calf Serum (Ca-FCS) or ovalbumin (OVA; Sigma), FCS or OVA was diluted in water to a protein concentration of 4 mg/ml. The diluted FCS or OVA was incubated for 12 hours at 37°C with 1 M potassium cyanate (Sigma-Aldrich). After incubation the sample was extensively dialyzed against water (as described before [14]). Citrullinated FCS (Ci-FCS) was generated by incubation of 10 mg FCS in a volume of 1 ml containing 0.1 M Tris-HCl pH 7.6, 10 mM CaCl_2_, and 40 U PAD4 (Sigma) for 24 hours at 37°C.

### Detection of Anti-CarP antibodies ACPA by ELISA

Non-modified FCS, citrullinated-FCS (Cit-FCS) and Ca-FCS were coated overnight at a concentration of 10 µg/ml (diluted in pH 9.6 0.1 M carbonate-bicarbonate buffer) on Nunc Maxisorp plates (Thermo Scientific). The plates were washed with PBS/0.05% Tween (Sigma) and subsequently blocked for 6 hours at 4°C with 100 µl of PBS/1% BSA (Sigma). After washing, the wells were incubated with 50 µl serum 1/50 diluted in PBS/1% BSA/0.05% Tween. The ELISA plates were incubated overnight on ice. Total Ig, IgG1, and IgG2a were detected using HRP-conjugated rabbit anti-mouse Ig antibody (Dako), HRP-conjugated goat anti-mouse IgG2a, HRP-conjugated goat anti-mouse IgG2c, HRP-conjugated goat anti-mouse IgG1 (all from Southern Biotec) or HRP-conjugated rabbit anti-human IgG (Dako). HRP enzyme activity was visualized using ABTS. As a standard, serial dilutions of a pooled serum sample from mice with CIA were used.

### Detection of Anti-CarP antibodies by immunoblotting

Equal amounts of FCS and Ca-FCS were loaded onto Hybond-C Extra membranes (Amersham). Blots were blocked for 1 hour with PBS/3% milk powder (ELK)/0.05% Tween for 1 hour at room temperature. The blots were washed six times with PBS/0.05% Tween. Serum from immunized C57Bl/6 mice was diluted 1∶200 with PBS/3% ELK/0.05% Tween. The blots were incubated with diluted serum for 1 hour at room temperature. After six washes with PBS/0.05% Tween, blots were incubated with HRP-labeled goat anti-mouse IgG2c diluted in PBS/3% ELK/0.05% Tween for 1 hour at room temperature. Next, blots were washed and bound antibodies were visualized using enhanced chemiluminescence (Amersham).

### Statistical analysis

All statistical testing was performed using Prism 5 (GraphPad Software). Outliers were determined using the Grubbs' test and excluded from the data analysis. Different groups of mice were compared using a Mann-Whitney U test. When multiple groups were compared a Kruskal-Wallis test was used followed by a Dunn's Multiple comparison test. Anti-CarP antibody levels over time were compared by calculating the area under the curve for each mouse, followed by a Mann-Whitney U test.

## Results

### Anti-Carp antibodies can be detected in mice

To detect anti-CarP antibodies, we developed an ELISA similar to the human anti-CarP antibody ELISA described previously [14]. Since we did not know whether mouse serum contained anti-CarP antibodies and which carbamylated proteins these antibodies would recognize, we opted to use a pool of different carbamylated antigens. It was important that this pool of antigens did not contain the antigen that was used for immunization, because this would make it difficult to determine the difference in antibody levels against the carbamylated and the non-carbamylated form of the protein. For these reasons, FCS was carbamylated (Ca-FCS) and used to coat the ELISA plates. Non-modified FCS was always included as a control to determine how much of the signal detected in the Ca-FCS coated well was specific to carbamylated proteins. As shown in figure 1B, serum from mice with CIA contains immunoglobulin's (Ig) that bind to Ca-FCS but not to non-modified FCS, indicating, for the first time, the presence of antibodies that specifically recognize carbamylated proteins in mice with CIA.

To further confirm the specificity of binding of these antibodies, we pre-incubated mouse serum containing anti-CarP antibodies with different concentrations of Ca-FCS and non-modified FCS. Following pre-incubation of the serum we performed an anti-CarP antibody ELISA. Pre-incubation with increasing concentrations of Ca-FCS blocked binding to Ca-FCS in a dose dependent manner, while pre-incubation of the sera with FCS did not affect the Ca-FCS binding (Figure 1C).

To further confirm the specificity of the antibodies using a different assay, we next tested the specificity of the antibodies by immunoblotting. Equal amounts of Ca-FCS and non-modified FCS were blotted on a nitrocellulose membrane. The presence of reactive antibodies in mouse sera was analyzed by incubating these blots with sera of four different immunized mice. On three out of four blots substrate developed on the location where Ca-FCS was blotted, but not on the location where FCS was blotted, indicating that serum from three out of four mice harbored antibodies specific for Ca-FCS (Figure 1D). The dot blot results were further reproduced with sera from an independent immunization experiment (data not shown). All tested serum samples that were positive in ELISA were also positive in these immunoblotting experiments. Together, these data indicate that anti-CarP antibodies are present in mice with CIA, and that their appearance does not require immunization with specific carbamylated antigens.

### Different isotypes of anti-CarP antibodies, but no ACPA, can be detected in mice with CIA

**Figure 2:**
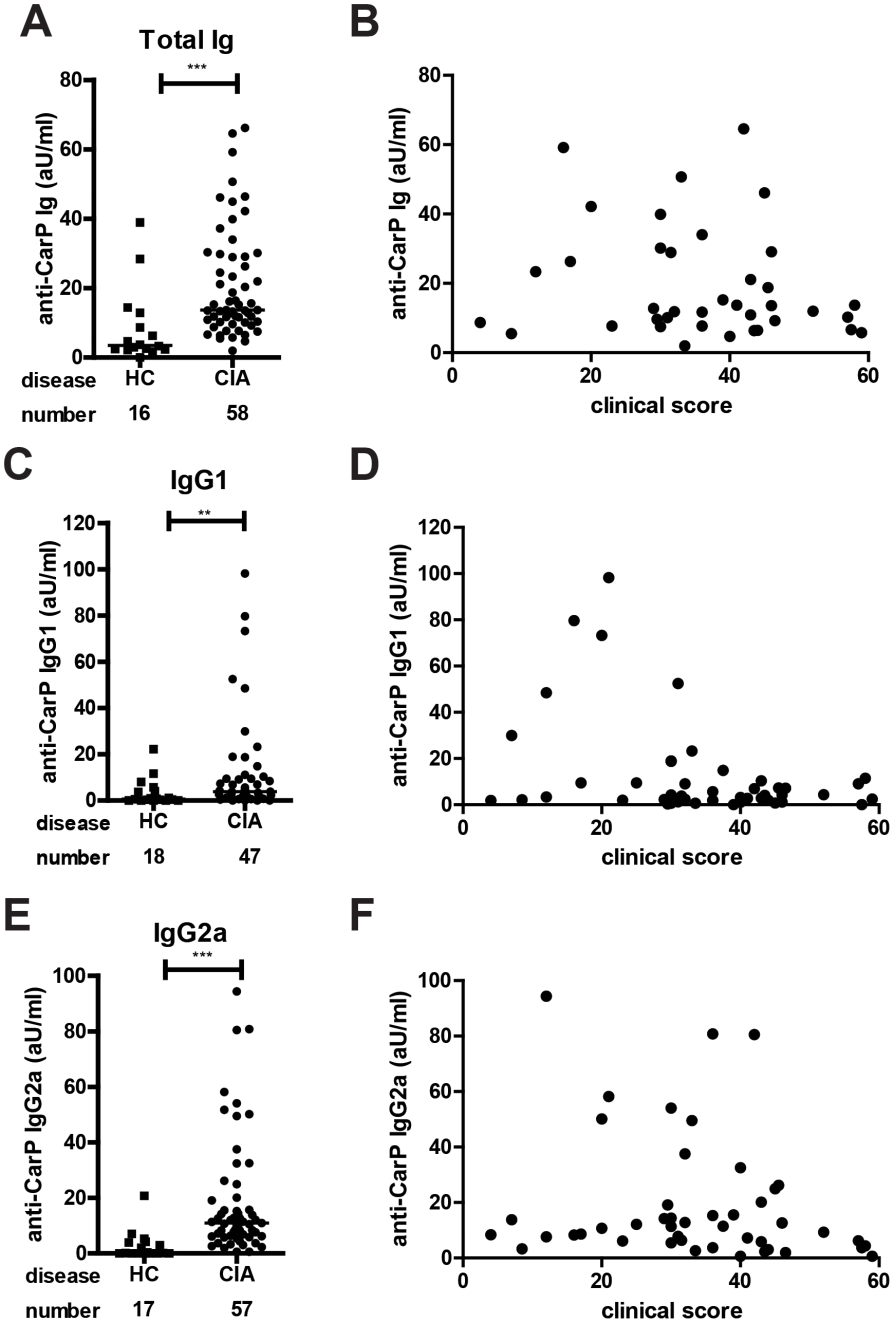
Mice with CIA harbor anti-CarP antibodies. CIA was induced in DBA/1J mice. After 70–90 days, serum of naïve non-immunized mice (HC; squares) and mice with CIA (dots) was harvested and anti-CarP levels were determined by ELISA. Arbitrary units were calculated using a standard curve of pooled serum from mice with CIA. The number indicates the number of mice per group. (**A**) Total Ig levels (**B**) Total Ig levels plotted against the clinical score (**C**) IgG1 levels (**D**) IgG1 levels plotted against the clinical score (**E**) IgG2a levels (**F**) IgG2a levels plotted against the clinical score. The data is pooled data from 4 independent experiments. Every symbol represents one mouse and the bar indicates the median. Statistical analysis was performed using a Mann-Whitney test (** p<0.01, *** p<0.001).

To determine whether the presence of anti-CarP antibodies in mice is related to arthritis, we compared anti-CarP antibody levels in the sera of mice with CIA and healthy non-immunized mice. Anti-CarP total Ig, IgG1 and IgG2a could be detected in mice with CIA, but not in non-immunized mice (Figure 2).

**Figure 3:**
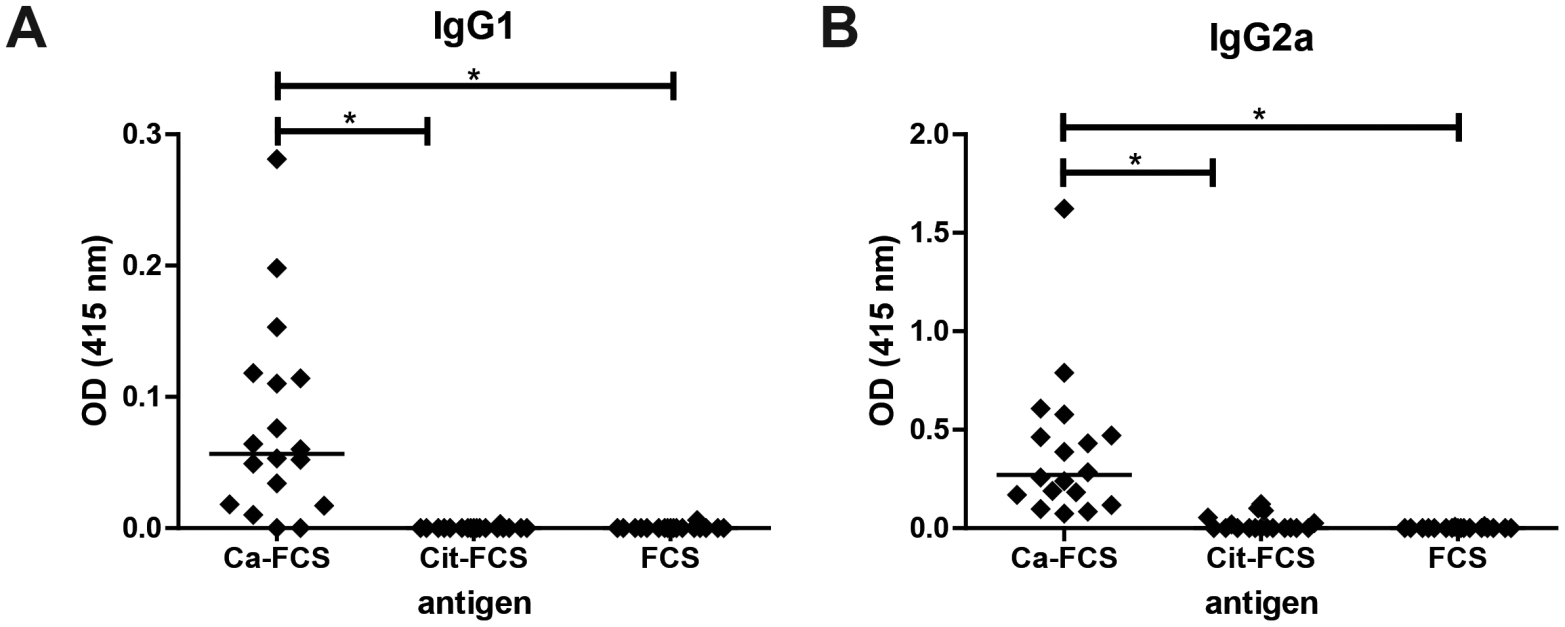
Anti-citrullinated protein antibodies (ACPA) could not be detected in mice with CIA. CIA was induced in DBA/1J mice. After 70–90 days, serum was harvested and anti-CarP and ACPA levels were determined by ELISA. Every symbol represents an individual mice and the line indicates the median. (**A**) IgG1 levels. (**B**) IgG2a levels. Statistical analysis was performed using a kruskall-wallis test followed by a Dunn's Multiple comparison test (* p<0.05).

We did not detect any antibodies that recognized citrullinated FCS in sera from healthy non-immunized mice or mice with CIA, indicating that there are no ACPA present in these sera (Figure 3). ACPA positive RA serum samples were used as a positive control in the ACPA ELISA. A validation experiment demonstrating the effectiveness of this method for detecting ACPA in CCP+ patient samples is depicted in Figure S1. These results are important as they indicate that mouse B cells discriminate between proteins containing homocitrulline and citrulline and they demonstrate it is unlikely that the reactivity towards carbamylated proteins we detect is caused by cross reactivity of ACPA.

Taken together, these results show that mice with CIA have different isotypes of anti-CarP antibodies, which we could not detect in healthy mice. Furthermore, the occurrence of isotype switching indicates a role for T cell help in the anti-CarP antibody response.

### The development of antibodies specific for carbamylated protein precedes disease onset in CIA

**Figure 4:**
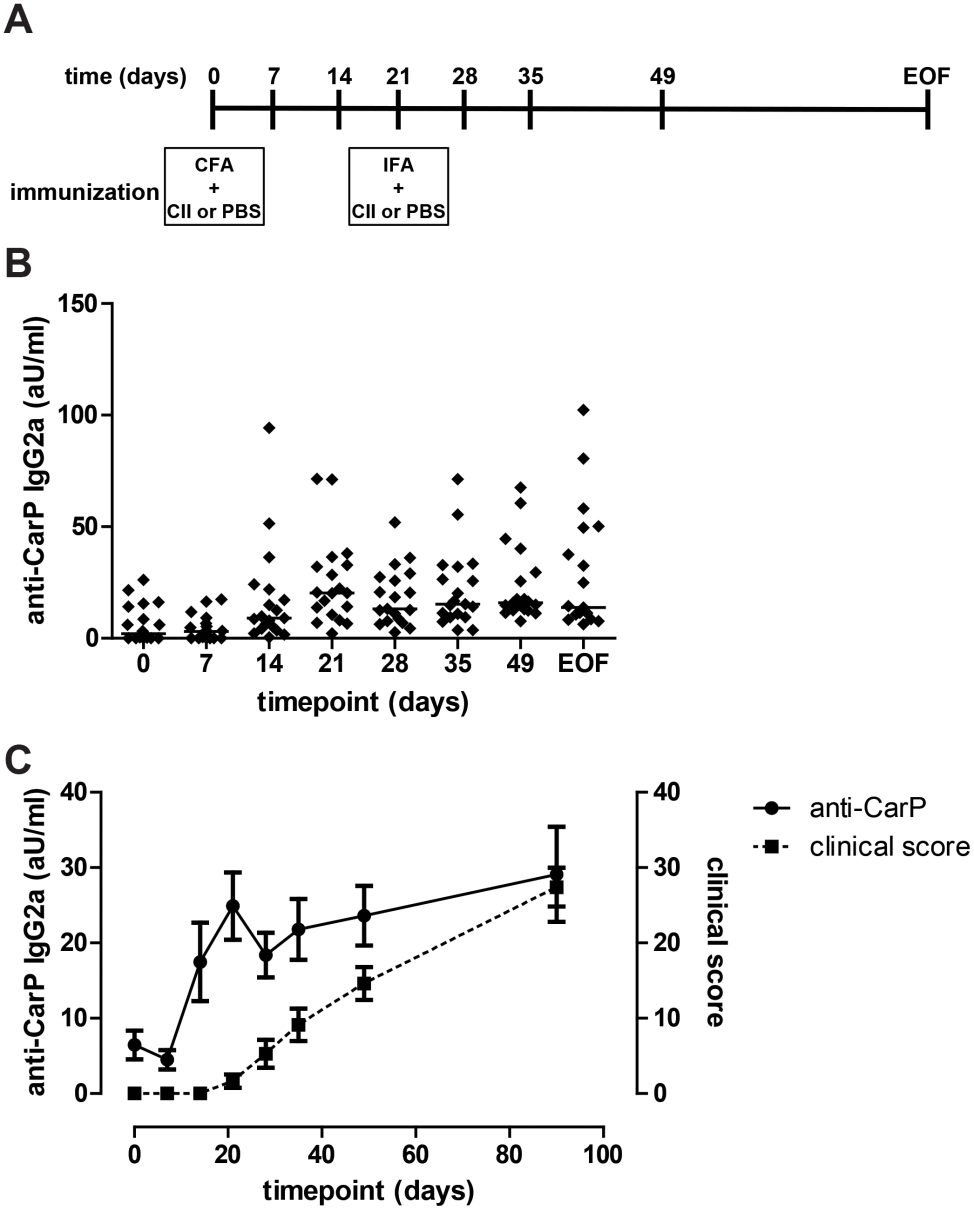
Kinetics of the anti-CarP response during CIA. (**A**) CIA was induced in DBA/1J mice (n = 19). The mice were immunized at day 0 and boosted at day 21. Blood was harvested from the mice at the indicated time points and serum was stored at −80. The anti-CarP antibody levels were determined in all serum samples simultaneously by ELISA at the end of the experiment. The data shown are the pooled data from 2 independent experiments that showed a similar trend. (**B**) Anti-CarP IgG2a levels were determined by ELISA. Arbitrary units were calculated using a standard curve of pooled serum from mice with CIA. Every symbol represents one individual mouse and the line indicates the median. (**C**) Anti-CarP IgG2a antibody levels are plotted on the left Y axes and indicated by the dots with the solid line. The clinical score is plotted on the right Y-axis and indicated by the squares and the dashed line. The error bars indicate the SEM.

After establishing that anti-CarP antibodies are present in CIA, we next investigated whether there was a relation between anti-CarP antibody levels and disease. We first studied the kinetics of the development of the anti-CarP antibody response. CIA was induced in DBA/1J mice and the mice were bled at different time points to monitor the development of the anti-CarP antibody response over time. From day 14 after immunization onwards, anti-CarP antibodies could be detected in the sera. The anti-CarP antibody levels increased until day 21 after immunization after which the anti-CarP antibody levels stabilized (Figure 4A). We observed an increase in serum anti-CarP antibodies before the onset of clinical symptoms (Figure 4B). We did not find a correlation between anti-CarP levels and the clinical score (Figure 2).

CII-specific IgG2a antibodies are important in the pathogenesis of CIA and their levels correlate with disease severity. Therefore, we compared CII-specific IgG2a levels with anti-CarP antibody levels. We did not find a correlation between the two antibody responses (data not shown). Together, these data indicate that the onset of clinical symptoms in CIA is preceded by an increase in serum anti-CarP antibody levels.

### Anti-CarP antibodies can be present independent of CIA

Because the incidence of CIA is >95% in DBA/1J mice, it is not feasible to determine whether development of anti-CarP antibody responses is specific for mice with arthritis/mice that will develop arthritis after immunization with CII in CFA. The CIA incidence is lower in C57Bl/6 mice. To study whether anti-CarP antibodies are specific for mice with CIA upon vaccination with CII in CFA, we next induced CIA in C57Bl/6 mice. In 2 independent experiments, anti-CarP IgG2c antibody levels could be detected in a proportion of the immunized C57Bl/6 mice; however their presence did not correlate with disease induction. Some immunized mice that did not develop CIA did also harbor anti-CarP antibodies. Similar to what we showed for DBA/1J mice (Figure 2), not all mice that developed CIA had detectable levels of anti-CarP antibodies indicating that the presence of anti-CarP antibodies in the circulation is not required for disease induction (Figure S2). Similar to what was observed for DBA/1J mice, we did not find a correlation between disease severity and clinical score (data not shown).

**Figure 5:**
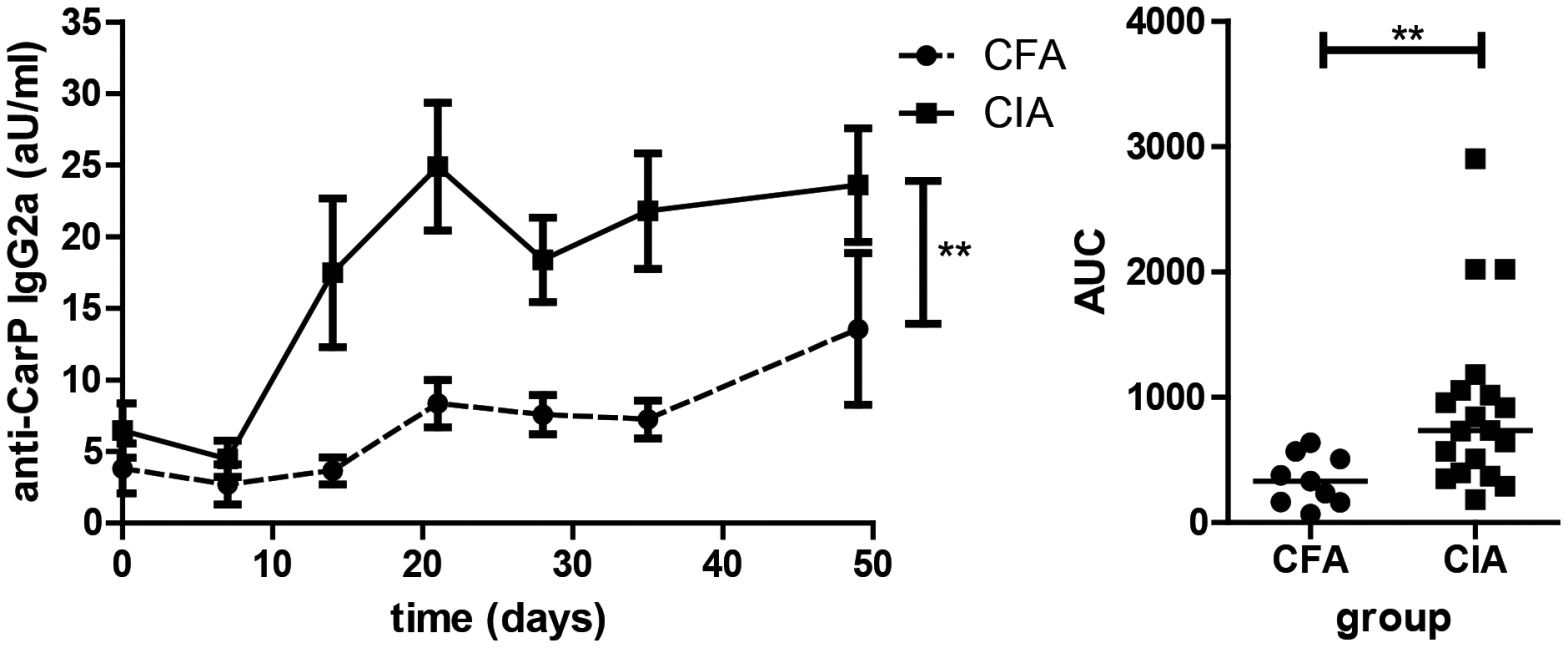
Anti-CarP can be detected in CFA immunized mice. DBA/1J were immunized with CII (CIA; (n = 19)) or PBS in CFA followed by a booster with CII or PBS in IFA (CFA; n = 9). Anti-CarP IgG2a levels were determined by ELISA. The data shown are the pooled data from 2 independent experiments that showed a similar trend. CFA +PBS immunized mice are depicted as circles and CIA mice are depicted as squares. The error bars indicate the SEM. In the right panel the area under curve is depicted. Every symbol represents one individual mouse and the line indicates the median. Statistical analysis was performed by comparing the area under the curve followed by a Mann-Whitney test (** p<0.01).

As we observed the presence of anti-CarP antibodies also in mice that did not develop arthritis and since immunization with CFA causes local inflammation, combined with the notion that inflammation can induce carbamylation [12], [13], we hypothesized that immunization of mice with CFA without an additional antigen can be sufficient for the induction of anti-CarP antibodies. To study this notion we determined anti-CarP antibody levels in DBA/1J mice that were immunized with CFA only (followed by a boost with IFA). We detected high levels of anti-CarP in three of these nine mice. Nonetheless, the anti-CarP antibody levels in mice with CIA increased earlier as compared to the CFA immunized mice. Over the course of the first 50 days after immunization the mice with CIA displayed significantly higher levels of anti-CarP as compared to CFA only immunized mice (Figure 5). Altogether these data indicate that immunization with CFA can lead to the induction of anti-CarP antibodies, however, the kinetics are different and magnitude of response is lower as compared to mice with CIA. More importantly, these data indicate that the induction of anti-CarP antibody responses is not directly related to arthritis or vaccination with specific carbamylated antigens, but instead, suggest that an inflammatory trigger can also lead to a break of B cell tolerance to carbamylated proteins.

## Discussion

A cornerstone of modern biomedical research is the use of mouse models to explore basic pathophysiological mechanisms. A key characteristic of one of the most common autoimmune diseases, rheumatoid arthritis, is the occurrence of antibody systems against post-translationally modified proteins. We now show that the mechanisms behind breaking of tolerance against post-translationally modified proteins, and in particular anti-Carp antibodies, can be studied in an animal model of arthritis. CIA is a mouse model of arthritis that shares several characteristics with RA and which is frequently used to study potential therapeutic strategies for RA [18], [19]. Antibodies play a key role in the disease pathogenesis of CIA [20]. Because of the importance of antibodies in CIA, this model is an interesting tool to further study anti-CarP antibody responses. In this study we show for the first time that, like in patients with RA, anti-CarP antibodies can also be detected in a proportion of the mice with CIA, however anti-CarP positivity does not necessarily correlate with arthritis, as we also detect the presence of anti-CarP antibodies in mice without arthritis.

In a previous study it has been reported that anti-CarP antibodies could be present in mice after immunization with CFA and a specific carbamylated peptide [16], but we now demonstrate that anti-CarP antibodies are readily detectable when mice are immunized with an emulsion of CFA and non-modified proteins (eg. the commercially available CII which is used for CIA induction). We show that immunization with CFA alone (without CII) can induce anti-CarP antibodies as well. Although we cannot exclude the possibility that CFA contains some proteins that are, at least partially carbamylated, our data indicate that there is no need to immunize with extensively carbamylated proteins or carbamylated peptides to breach B cell tolerance leading to the emergence of anti-CarP antibody responses. We consider it likely that anti-CarP-Immune responses can occur resulting from inflammatory conditions induced by for example infection, as it is conceivable that such conditions lead to the carbamylation of pathogen-derived proteins or self-proteins.

We have detected different anti-CarP isotypes and subclasses. For most B cell responses T cell help, in the form of cytokines and CD40-CD40L ligation, is required for isotype switching to occur [21]. Therefore, the fact that isotype switching occurs indicates a role for T cell help in the anti-CarP antibody response.

It was recently shown that ELISA can give false positive results when determining ACPA levels in mouse serum and it was suggested that immunoblotting represents a more reliable technique [10]. To exclude the possibility that we obtained false positive results in the anti-CarP antibody ELISA, we also verified the specificity of the antibodies by dot blot. In these assay's we only detected binding to Ca-FCS and not to FCS confirming the results obtained by ELISA. Since the immunoblotting assays and the ELISA gave similar results, we used ELISA for all the other experiments because it is a more high-throughput method and it allows for more reliable quantification. To further show the specificity of the anti-CarP antibodies we performed inhibition assays. Pre-incubation of the sera from mice with CIA with increasing concentrations of Ca-FCS could block Ca-FCS binding in the ELISA, while pre-incubation with non-modified FCS had no effect. The increased binding of sera to Ca-FCS cannot be explained by differences in the amount of protein that was coated on the ELISA plate. Experiments with Ca-OVA and non-modified OVA revealed that carbamylated proteins are not binding better to ELISA plates (data not shown).

Although citrullinated FCS can readily be used to demonstrate the presence of ACPA in human anti-CCP positive sera (Figure S1) we could not demonstrate the presence of ACPA in the context of the study presented. Mice with CIA can have citrullinated antigens in their inflamed tissue [9], however, no ACPA could be detected in mice with CIA indicating that the B cell tolerance to citrullinated antigens was not broken in our experiments. Nonetheless, we detected the presence of anti-CarP antibodies in most arthritic mice analyzed indicating that B cell tolerance to carbamylated antigens is broken more easily as compared to the B cell tolerance to citrullinated antigens. Whether carbamylated proteins are present in arthritic tissue will have to be addressed in future studies.

The appearance of anti-CarP antibodies in the serum before disease onset is similar to what we have found in patients [22]. Anti-CarP antibodies can be detected in serum of arthralgia patients before the onset of RA [15]. The observations that a large part of the mice with CIA harbor anti-CarP antibodies, that these antibodies precede the onset of clinical disease and that anti-CarP antibodies can be found in non-arthritic mice, nicely recapitulates the current findings in humans.

We did not find a correlation between anti-CarP levels and clinical score in our mice. However, this could be explained by the fact that the clinical score predominantly monitors the level of inflammation. Furthermore, in RA patient samples we did also not detect a correlation with the inflammation, but only with radiographic damage [14]. This issue can potentially be addressed in future studies using micro-CT imaging.

The onset of the anti-CarP antibody response during CIA is more or less similar to what has been reported for the onset of the anti-CII antibody response, since both antibodies can already be detected 2 weeks after immunization [23]. However, while anti-CII antibody levels still increase after 21 days [23], the anti-CarP antibody levels did not. Together, these findings indicate that the kinetics of the anti-CarP response are different from the anti-CII response.

Not all mice that develop CIA had detectable levels of anti-CarP antibodies in their serum, indicating that unlike CII-specific antibodies, anti-CarP antibodies are not required for disease induction. We could also detect anti-CarP antibodies in serum of immunized C57Bl/6 mice that did not develop CIA and in DBA/1J mice that were immunized with CFA (without CII). Similar observations have been made for anti-CII antibodies in C57Bl/6 mice, where after immunization all mice had anti-CII antibodies, however not all mice developed arthritis [24]. Since anti-CII antibodies are considered to be pathogenic, but can be present in non-arthritic immunized mice, the presence of anti-CarP antibodies in non-arthritic mice does not exclude the possibility that anti-CarP antibodies can play a role in disease pathogenesis as well. The potential pathogenicity of anti-CarP antibodies will have to be addressed in future studies with e.g. anti-CarP monoclonal antibodies in a similar fashion as has been described before for ACPA [7], [10].

In conclusion our results indicate that immunization with CFA can lead to a break in tolerance to carbamylated antigens, resulting in the induction of a specific antibody response and that this anti-CarP antibody response is stronger in mice with arthritis compared to immunized control mice. The presence of anti-CarP antibodies in CIA indicates that the CIA model could function as an in vivo model to study anti-CarP responses.

## Supporting Information

Figure S1 **Citrullinated-FCS can be used to detect anti-CCP in human sera.** Human sera was divided in 2 groups based on the anti-CCP status. The anti-Cit-FCS Ig levels were determined by ELISA. Every symbol represents 1 serum sample and the line indicates the median.
(TIF)

Figure S2 **Anti-CarP antibody levels in C57Bl/6 mice.** CIA was induced in C57Bl/6 mice in two independent experiments. After 70–90 days, serum of naïve non-immunized mice (squares) and immunized mice that did not develop CIA (dots) and immunized mice that developed CIA (diamonds) was harvested and anti-CarP levels were determined by ELISA. The left panel shows the first experiment and the right panel shows the second experiment.
(TIF)
