# Supplementary figures and images for: Antibodies Specific for Carbamylated Proteins Precede the Onset of Clinical Symptoms in Mice with Collagen Induced Arthritis
Source: PLoS One. 2014 Jul 15;9(7):e102163. doi: 10.1371/journal.pone.0102163 (PMC4099068; doi:10.1371/journal.pone.0102163)

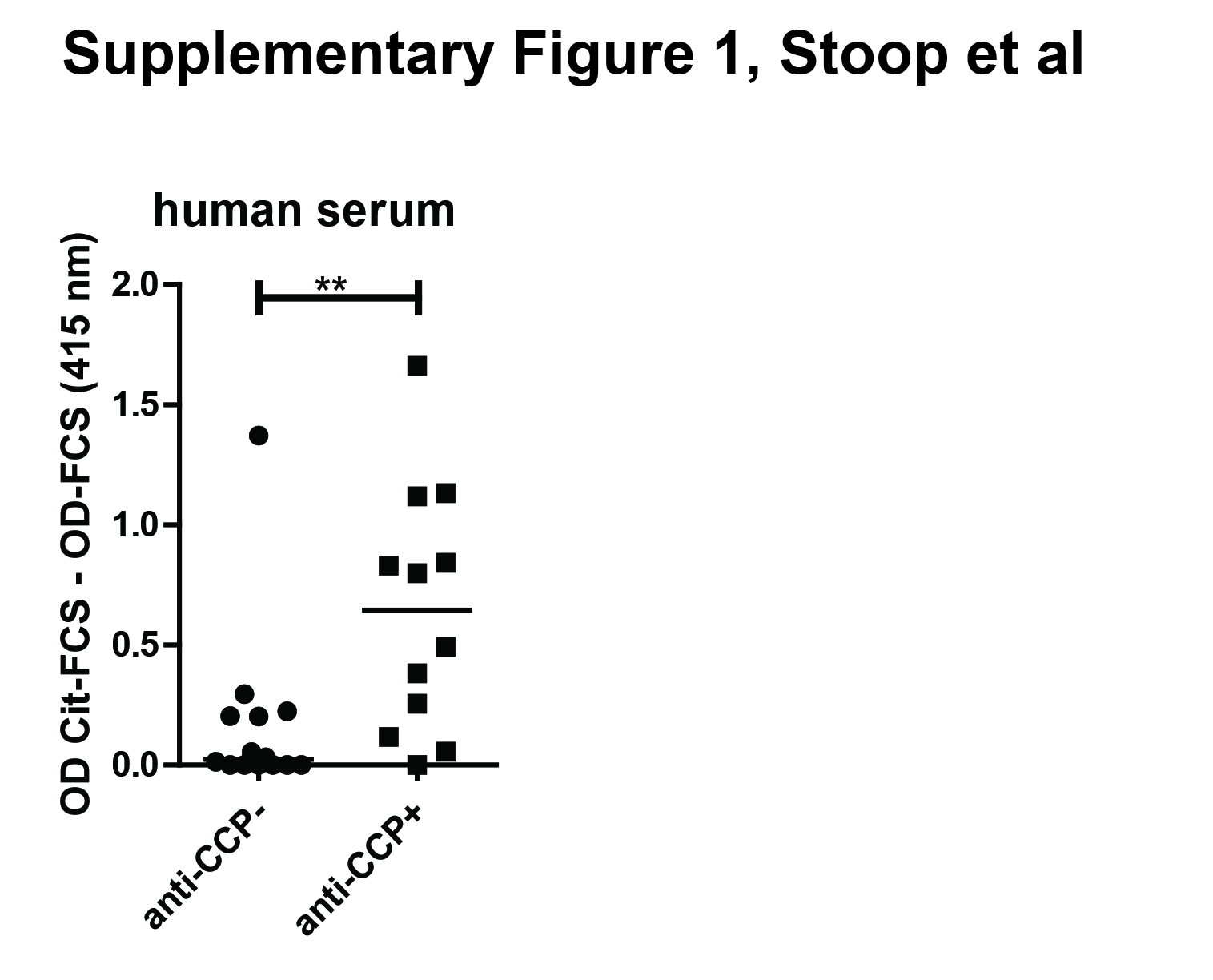

Supplement: Figure S1 — Citrullinated-FCS can be used to detect anti-CCP in human sera. Human sera was divided in 2 groups based on the anti-CCP status. The anti-Cit-FCS Ig levels were determined by ELISA. Every symbol represents 1 serum sample and the line indicates the median. (TIF) [file pone.0102163.s001.tif]

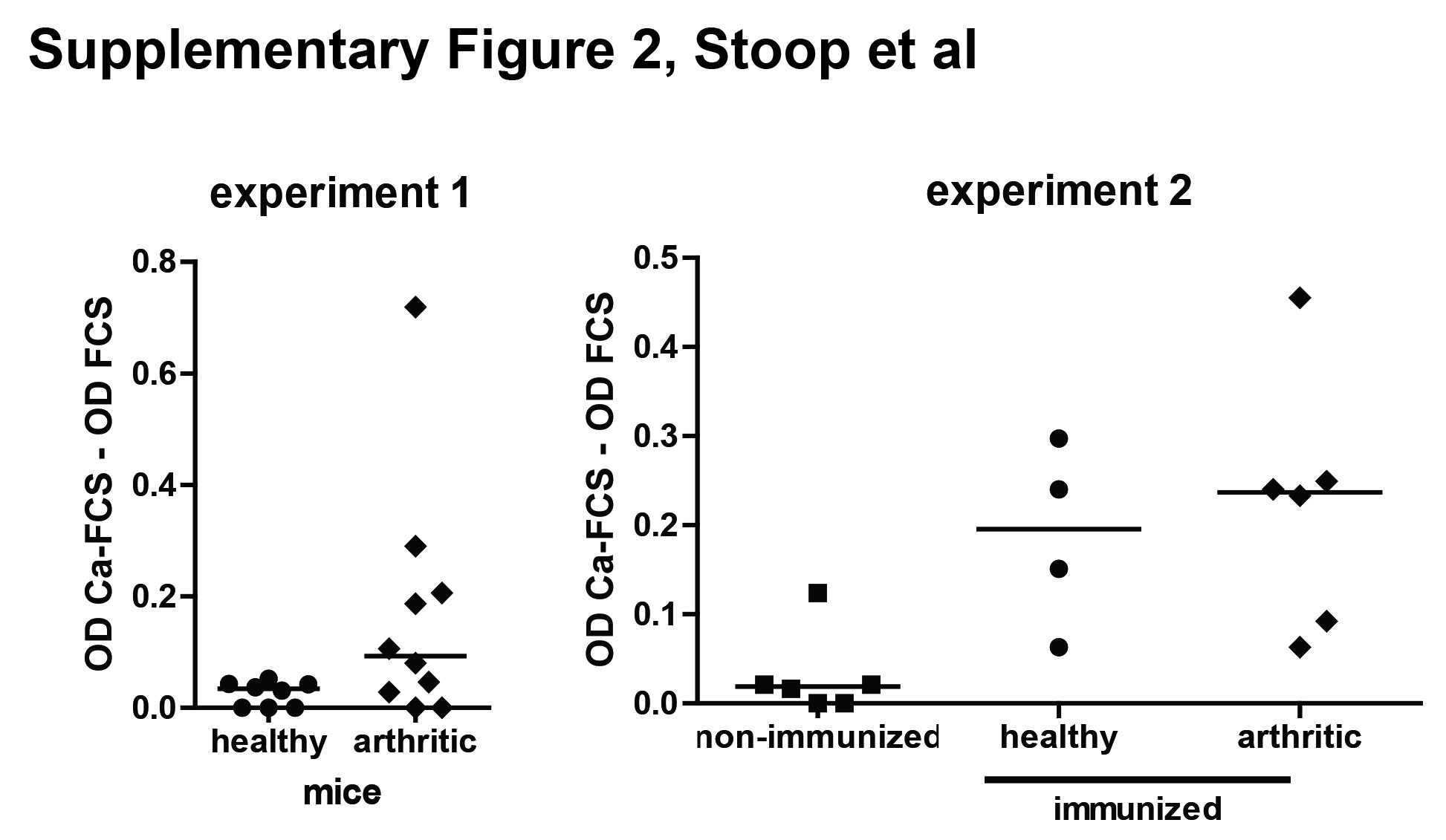

Supplement: Figure S2 — Anti-CarP antibody levels in C57Bl/6 mice. CIA was induced in C57Bl/6 mice in two independent experiments. After 70–90 days, serum of naïve non-immunized mice (squares) and immunized mice that did not develop CIA (dots) and immunized mice that developed CIA (diamonds) was harvested and anti-CarP levels were determined by ELISA. The left panel shows the first experiment and the right panel shows the second experiment. (TIF) [file pone.0102163.s002.tif]
